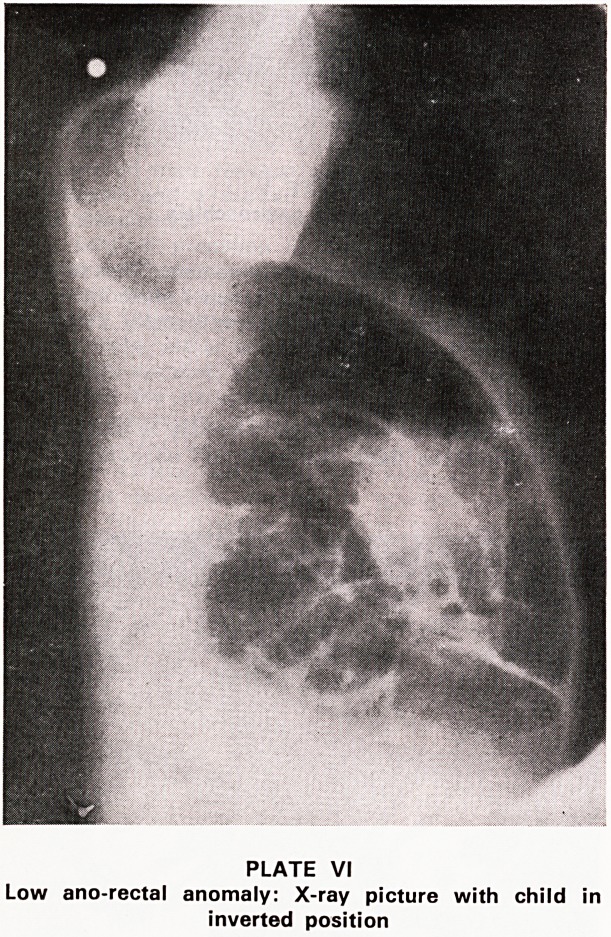# Some Surgical Problems of the Newborn
*A Paper read to Bristol Medico-Chirurgical Society.


**Published:** 1974-04

**Authors:** A. G. McPherson

**Affiliations:** Consultant Surgeon, Southmead Hospital, Bristol


### Some Surgical Problems of the Newborn*

A. G. McPHERSON, M.Ch., F.R.C.S.

Consultant Surgeon, Southmead Hospital, Bristol

One might ask why a general surgeon, most of whose work concerns adults, many of them at the opposite extreme of life, should have chosen to speak about the surgery of the newborn. There are, of course, many reasons.

Southmead Hospital, with its large Obstetric and Paediatric units and in particular the Special Care Baby Unit, has provided the material and the stimulus for me to develop, over the years, a special interest in those aspects of paediatric surgery which I consider to be within the province of general surgery. There is a great deal of team work in this, as in other branches of medicine, and I am fortunate in having available not only skilled paediatricians and nursing staff, but also the services of pathologists and radiologists with great knowledge and interest in neonatal conditions.

One might also question whether, since a considerable degree of specialisation is involved, this work should be more properly done by 'Pure Paediatric Surgeons' or continue, as in Bristol, to be carried out by general, neuro-, orthopaedic, plastic, thoracic and urological surgeons (the order is strictly alphabetical) who have a special interest in paediatrics but who also possess the particular skills of their own specialty. After all they can co-operate with one another, as need be, and lean as heavily as they may wish on the paediatrician's expertise in baby management.

**History**

All known clinical medicine goes back to Hippocrates (460–373 B.C.), and some of his aphorisms have a paediatric-surgical if not exclusively surgical slant. I quote: 'The surgical diseases of the new born and infants are aphthae, vomiting, night fears, inflammation of the umbilicus and discharge from the ears. A little later there are tonsillar affections, crick in the neck, asthma, calculus, round worms, scrofula, tumours about the ears and elsewhere' and the last aphorism: 'Those who acquire a Gibbus spine with cough and asthma before puberty die.'

Skipping two millenia we come to Thomas Phaer (1510–1560), the 'Father' of English paediatrics. His 'Boke' is of historic rather than surgical interest. Nevertheless, at or about this time the great German physician, Paracelsus, specialised in removing bladder stones from children. The clue to why he called himself Paracelsus may reside in his real name—Theophrastus Bombast von Hohenheim!

An intimate of Paracelsus was Felix Wurtz (1518–1574). He described himself as a surgeon and inspired 'The Children's Book', which contained in it his expert treatise, mainly directed at surgeons, midwives and wet nurses, at a time when infant mortality was appalling, and much of it preventable, due to the wilful ignorance of those dealing with children. To us today much of it would seem simple common sense. He pointed out the necessity for gentle handling, and how to recognise an ill child. He indicated practices which were common but harmful. For example, he wrote 'Children are hurt if after bathing they are laid behind a hot oven'. Nancy Mitford in her book 'The Sun King', paints a gloomy picture of medical practice in those days and firmly, if cynically, places much of the blame for infant mortality upon the doctors.

Guy Fagan (1638–1718), was Louis XIV's Principal Doctor, and his fame extended all over Europe. In twenty years he managed to see most of the French Royal Family into their graves, including, from misfortune and mismanagement, three generations of Louis XIV's heirs, within a period of eleven months. Quoting from Miss Mitford: 'It was common practice in those days that, when a child was ailing, first they (the doctors) bled it, then purged it, and then administered an emetic—which generally did the trick'.

This was more than a century after the publication of Felix Wurtz's treatise. But things were not so black everywhere; children's medicine, and surgery, though haltingly, were progressing. Francis Glisson (1597–1677), published his treatise on Rickets in 1650, four years before his better known Anatomia Hepatis Wolfgang Höffer (1614—1681), noting the frequency of foolishness and struma in the Alps first described cretinism. The first tracheostomy was performed by Pierre Bretonneau (1777–1862). Orthopaedics emerged due to Nicholas André of Paris (1658–1742), and the origin of paediatric surgery, as a speciality, was due to Jean Mathieu Delpech of Montpelier (1771–1822).

Skipping a few centuries, we come to the present one, when the cumulative discoveries of the past were overtaken by an explosion of new scientific knowledge in many fields, which, *inter alia,* enormously increased the scope and safety of neonatal surgery. Here one must mention Denis Browne (1892–1967), of whom it has been said that he was the acknowledged Father of Paediatric Surgery in the English speaking world. In America Robert E. Gross of Harvard (b. 1905), was responsible for placing paediatric surgery on a firm basis and his great book 'The Surgery of Infancy and Childhood', published in 1953 covering in great detail the whole range of paediatric surgery, as it had so far developed, became as a Bible to those who found themselves involved in such surgery.

I should like now to deal with some specific clinical problems, and should state that my interest and experience has been mainly but not exclusively cor

* *A Paper read to Bristol Medico-Chirurgical Society.*

cerned with problems of the abdomen or its parietes in the neonate.

**Pyloric Stenosis**

In the abdomen I will start with a condition with which you are all familiar, since it is relatively common, namely Congenital Hypertrophic Pyloric Stenosis.

The condition was first described by Patrick Blair in 1717, but prior to 1912 surgical treatment, which was by gastro-jejunostomy, was almost universally fatal. In that year Conrad Ramstedt, attempting formal pyloroplasty and trying to suture the divided muscle found that the sutures cut out and closure was incomplete. In his next case he made no attempt to suture the muscle but found the result to be equally good. Since then this delightfully simple operation, which could be tolerated at a period when anaesthesia and supportive treatment were fairly primitive, has become the standard treatment and under the conditions of today carries virtually no mortality.

**Other Congenital Anomalies**

It is only since the Second World War that it has been possible to operate on neonates and premature babies with severe anomalies, requiring very major surgery with an acceptable rate of survival. In fact the problem presented nowadays is sometimes quite a different one, namely how far is it ethically and morally justifiable to salvage, by sophisticated and often costly team work, infants who *may* live, but who by the nature of their residual disability may be gravely handicapped and a burden to the family, to society and to themselves?

The surgical problems in the neonatal period are quite distinct from those of adult life and one is dealing almost exclusively with the pathology of congenital anomalies. These can affect any system and are so often multiple, either within one system, or involving two or more systems, that one should regard the single anomaly as the exception rather than the rule.

A minor associated anomaly may be unimportant but when there are two or more major anomalies, or other unfavourable factors, survival rates are poor and one should question whether it is right always to interfere. When, however, there is only one major anomaly threatening life, survival after surgery is becoming the rule and, more important, the quality of life saved is often good.

There are some general points to stress when considering major surgery in the neonate. Many of these babies are small or premature. Birth itself is a traumatic experience. Nevertheless, the neonate will tolerate a great deal of added surgical trauma provided the necessary skills are available. These include the expertise of the modern anaesthetist and a high standard of paediatric and nursing care. Adequate biochemical monitoring is also required.

Haemorrhage is poorly tolerated so a reliable intravenous infusion must be in place and blood must be available and be given to replace blood loss as it occurs. It may be necessary to maintain fluid and electrolytic balance for a long period by the intravenous route.

Chilling, particularly of the premature baby, must be avoided. This is easier said than done. Temperature should be monitored when there is this risk. An intratracheal catheter is essential.

A generous abdominal incision should be employed whenever necessary. Only by adequate exposure can the precise nature of many intra-abdominal anomalies be diagnosed and accurate diagnoses must precede correction.

The true incidence of congenital anomaly is difficult to ascertain and numbers expressed per thousand live births are difficult to comprehend.

Numerically the problem is small, pyloric stenosis being about twice as common as all the other neonatal abdominal problems put together. Spina bifida with hydrocephalus is also twice and congenital heart disease three times as common. At the same time no two cases present an identical problem. For this reason alone it would seem desirable to concentrate the cases to special units and to a few surgeons only, so that sufficient experience may be acquired.

Over the years I have dealt with about 120 neonatal small bowel obstructions, fifty anorectal anomalies, and a mere twenty or so neonatal perforations. It is fair to point out that latterly the numbers referred for surgery have been steadily rising and are likely to continue to rise with increasing specialisation and centralisation of special care facilities.

Some problems are obvious on external examination

**PLATE III**
**Large exomphalos**

of the baby, although the precise nature and treatment are not always so obvious.

**Abdominal Wall Defects**

Defects of the abdominal wall are exomphalos where there is a herniation into the cord or the amniotic sac, and the much rarer gastroschisis where the intestines eviscerate through a paraumbilical defect, usually being matted together by a foetal peritonitis.

With a small exomphalos one-stage closure presents no difficulty. In other cases there is too large a defect and too small an abdominal cavity so that a staged closure is required. In the first stage skin closure only is attempted. As the child grows the defect becomes relatively smaller, and secondary repair becomes possible. Sometimes the discrepancy is extreme and skin closure is achieved with great difficulty.

The respiratory embarrassment consequent on restoring virtually all the bowel into the tiny abdomen is a real problem and with the present availability of new materials, I would no longer attempt it.

A recent case shows a large exomphalos (Plate III) where the defect was closed with silastic sheet (Plate IV) sparing the anaesthetist from all problems of respiratory embarrassment. Technically it was not a complete success and the silastic, in which it was planned to take successive tucks, was abandoned after ten days and 2% Mercurochrome applications substituted. Closure was complete at three months but later he will require a secondary repair of the musculature.

PLATE IV
**Exomphalos: closure with Silastic**

Even with a small defect the exomphalos may rupture and peritonitis may ensue. A much more serious condition is gastroschisis (Plate V) where there may be evidence of ante partum faecal peritonitis. Some such cases also show exstrophy of the cloaca. There is an exomphalos but in addition there is a gross maldevelopment of the lower abdomen and perineum, and there is no anal or genital development. This forms a convenient bridge to the next group I shall introduce, that of Ano-Rectal Anomalies traditionally known as 'Imperforate Anus'.

PLATE V
**Complicated gastroschisis**

**Ano-Rectal Anomalies**

Classification of Ano Rectal Anomaly is difficult due to the enormous individual variation encountered.

FIG. 1
**Varieties of ano-rectal anomaly**

The early classification of Ladd & Gross into four groups has been superseded and a suggested international classification offers *28 types* which seems to me to be excessive. Something simpler appeals to me as being of more practical use.

First of all, about 5% have a normally sited anus and it is usually stenosed rather than totally imperforate. These are the simplest of the anomalies to treat and dilatation alone will usually be all that is required.

The majority of cases have no anus at the correct site, and most of these have an ectopic opening or a potential fistula, and I find it helps to regard them as cases of ectopic anus.

Figure 1 clearly indicates that the most important factor in these anomalies is whether the anomaly is high or low. This is fundamental; to treat a low anomaly mistakenly as a high one is unpardonable. If the ectopic opening is low, as in the upper diagrams, then the rectum has developed below the levator ani, and there is at the very least an intact pubo-rectalis sling, which is the key to continence.

**PLATE VI**
**Low ano-rectal anomaly: X-ray picture with child in inverted position**

A simple surgical procedure is all that is usually required.

In the high cases, there is ano-rectal agenesis and the bowel ends above the levators; an opening if present will communicate with the genital apparatus, usually the vagina, in the female, or with the urinary tract in the male. A major operation is required and continence is likely to be very much less than perfect.

Lateral X-rays in the inverted position (Plate VI) may be helpful but must be interpreted with care. The lateral view alone gives information. Rectal gas very close to the skin marker will prove a low anomaly, but such a picture will not be shown in the first few hours after birth; it may be 24 or more hours before swallowed air has reached this point. All my cases of low anorectal anomaly have been simple to treat and are continent of faeces. But at this point I should like to emphasise that associated anomalies in the upper urinary tract are common and not restricted to cases with high anomaly. All cases, high or low, require a pyelogram to exclude this at some stage of their management.

In the case of a high anomaly the bowel terminates above the levators ani and at a considerable distance from the anal dimple. In the male intestinal obstruction is the rule, so that surgery is of some urgency. The high anomaly is less common in the female where there is usually a cloacal arrangement. In extreme cases the bowel opens high into a single passage which must serve as vagina, rectum and urethra. In the female obstruction is usually absent in the high anomalies and surgery is not urgent.

Treatment of high anomalies requires a major 'pull-through' abdomino-perineal procedure. In the past it has been my policy to do a one stage procedure whenever possible as soon as the diagnosis of a high anomaly has been confirmed. Provided there are no additional major anomalies, infants now regularly survive this procedure and one avoids a colostomy which has a high complication rate in small babies. Rectal continence is never perfect, since not only is there no proper sphincter apparatus, but in addition there is a lack of normal ano-rectal sensation. Nonetheless, with training of bowel habit and encouraging voluntary contraction of the levator ani, through which the bowel has been brought, an acceptable situation has usually been achieved. Sometimes conflict may develop between mother and child and contribute to a poor functional result, but I would like to stress that where the parents accept the situation, are intelligent, tolerant and affectionate, the results are good.

In one case, at the age of ten, with the help of Mr. Harry Griffiths, I performed a Gracilis transplant round the anal canal with an electrical implant. The smaller mechanism is buried. An induced current from the larger external part stimulates the implant. This action is entirely voluntary and six months later, he had ceased to use the stimulator.

(A short film illustrating this method of treatment was shown at this point.)

**Neonatal Intestinal Obstruction and Perforation**

Neonatal obstructions and perforations form a larger and very mixed group and present much greater diagnostic difficulty, which can only be fully resolved at laparotomy.

They usually present with bilious vomiting, abdominal distension and failure to pass meconium.

The importance of vomiting of bile cannot be overstressed, and always demands investigation. It frequently indicates a surgical emergency, but as some cases are of a functional nature and can be managed conservatively, full investigation must precede surgery.

The key to the problem is often the radiography and the interpretation of the X-ray findings. Plain films are often sufficient to confirm that surgery is required but cannot be expected to determine the site or precise nature of the obstruction in many cases. Serial plain films often help and in the hands of an experienced radiologist contrast studies, with radio-opaque meal and follow through or enema may be most valuable and safe.

The commonest site for atresia or stenosis is the duodenum, and the plain film is often diagnostic. Another cause of incomplete obstruction is by single or multiple transverse diaphragms in the gut. Less common but of special interest are obstructions due to malrotation. It interested me to find that in 1923 Norman Dott, later of neuro-surgical fame, published a paper describing in detail the errors of rotation. Two of his three cases were neonates who died of malrotation and volvulus, the true nature of the operative findings not having been appreciated in those days.

Errors of rotation are associated with lack of normal fixation of the bowel so that the whole midgut, suspended on a narrow mesentery, may easily undergo a volvulus, but also abnormal adhesions may occur, often across the duodenum (Ladd's bands) which themselves may cause obstruction. It is essential to divide these bands in addition to untwisting any volvulus, if it has occurred.

Another not uncommon cause of small bowel obstruction is meconium ileus. This is associated with fibrocystic disease of the pancreas and a generalised mucoviscidosis. Sometimes the plain X-ray films are diagnostic showing small bowel distension, without fluid levels and a 'soap bubble' appearance. It may be complicated by volvulus or perforation, and if so the prognosis is extremely bad.

Organic obstructions of the large bowel, apart from ano-rectal anomalies, are rare.

**Hirschsprung's Disease**

In 1886 Harald Hirschsprung described the clinical and autopsy findings in two infants dead of constipation with dilatation and hypertrophy of the colon. Although not the first to describe the condition, he was the first to recognise that a congenital malformation might be the cause.

In 1898 Sir Frederic Treves attributed the cause to congenital spasm of the distal segment and although as early as 1901 several authors had described degeneration in the cells of Auerbach's and Meissner's plexuses in the apparently normal distal colon, it was to be many years before Swenson & Bill in 1948 showed conclusively that it was not the proximal dilated and hypertrophied colon that was at fault, but that this was secondary and due to absence of movements in the distal narrow segment. On this they based their curative operation of rectosigmoidectomy. In the same year two papers showed the condition to be due to aganglionosis. This was subsequently confirmed by Martin Bodian, among others, who drew attention to an important positive finding, that of abnormally large nerve plexuses in the place of ganglia.

The mortality of this disease was extremely high until its nature and a rational surgical approach became known, but it is not always easy to diagnose in the early neonatal period. Except in the milder cases surgery is necessary. In the neonatal phase proximal colostomy alone should be attempted, resection being postponed until later.

**Perforation of the Gut in the Newborn**

Finally the twenty neonatal perforations which I have encountered, emphasise the enormous individual variation of neonatal disease. In addition to maternal hydramnios in five cases and prematurity in seven, there were in this small group, with some overlap, twelve different aetiologies.

Four cases had atresia and strangulated volvulus, and there were four perforations of the colon associated with exchange transfusion for haemolytic disease.

Three cases had fibrocystic disease of the pancreas.

Two or possibly three cases had congenital deficiency of the bowel wall. In one case the affected patches of bowel wall were reduced to peritoneum only and tissue paper thin, and had ruptured in places.

There were single cases of perforation from Hirschsprung's disease, gangrenous intussusception, Meckel's diverticulum and appendicitis, and one each of tubular intestinal duplication, and a ruptured enterogenous cyst.

**Conclusion**

In the neonatal abdomen one must expect to find practically anything.

I have illustrated some of the varied and difficult problems presented in this group of neonatal abdominal emergencies. There is great satisfaction when surgery meets with success, as for instance a little lass who survived operation for meconium ileus and continues to defy the usual poor prognosis associated with the disease, and also a young lady who had two surgical interventions in the neonatal period to become, as you see her here, beautiful, healthy and contented.

In conclusion I would like to thank all my colleagues who have helped me with these problems and would add a special mention of Mr. W. G. Sweet for the photography.

## Figures and Tables

**PLATE III f1:**
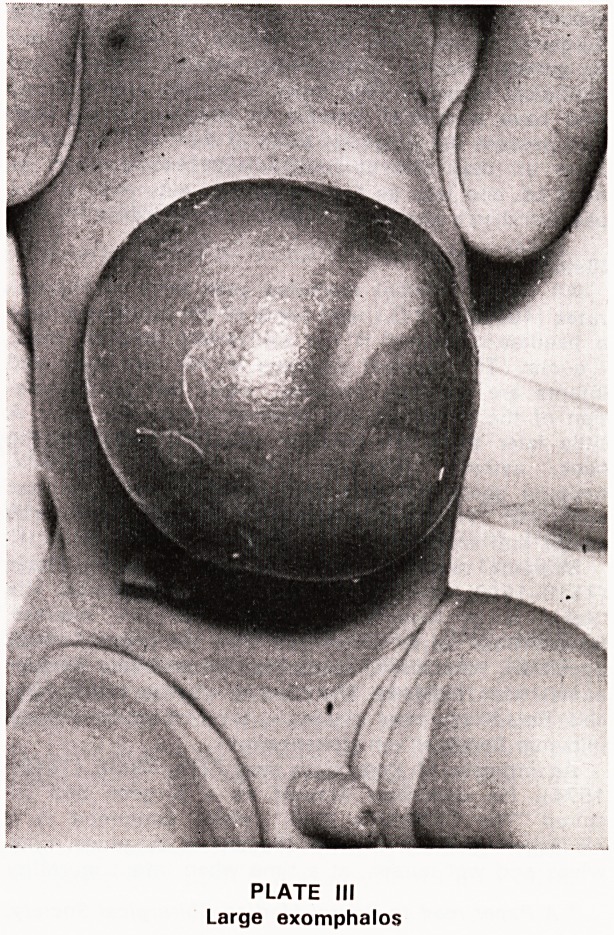


**PLATE IV f2:**
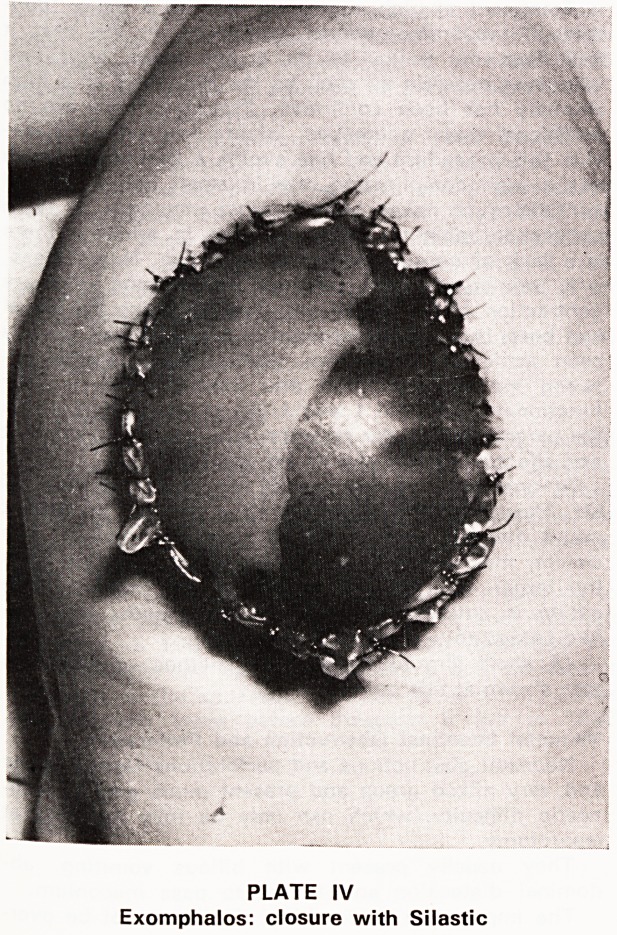


**PLATE V f3:**
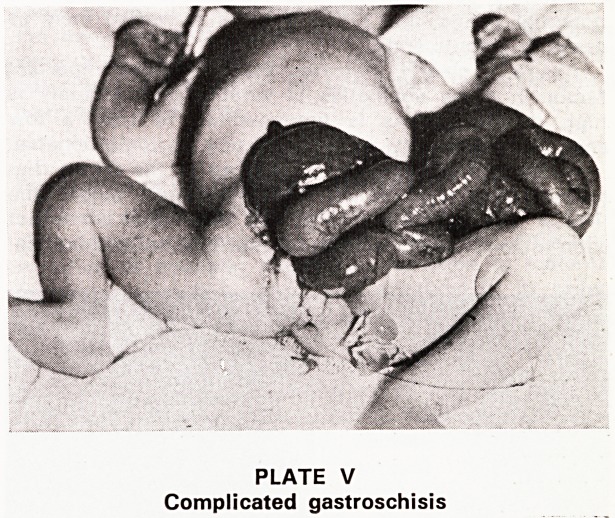


**FIG. 1 f4:**
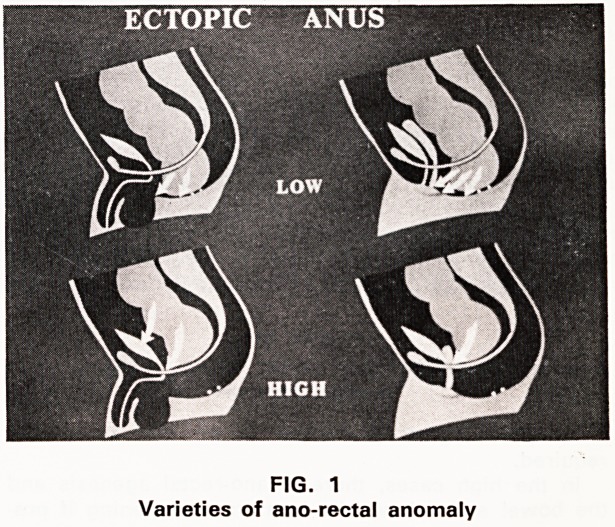


**PLATE VI f5:**